# Association of Faculties of Medicine of Canada response to: The unmatched by Dr. Amit Persad

**Published:** 2018-05-31

**Authors:** Geneviève Moineau

**Affiliations:** 1 Association of Faculties of Medicine of Canada / L’Association des facultés de médecine du Canada, Canada

Dr. Persad eloquently describes the extraordinarily difficult circumstance of the unmatched Canadian medical graduate (uCMG). We have heard many students describe the incredible sense of loss, and the significant reduction of self-confidence they experience. They have also reminded us of the stigma of being unmatched that continues to exist in our environment.

What is important for everyone to understand, particularly those who do not match, is that most unmatched graduates of Canadian medical schools are deemed competent by their medical school and eligible to receive their medical degree. We know that in 2018, 78% of the unmatched students were ranked by a residency program. In other words, if a residency position had been available, they would be starting their residency on July 1^st^.

This clearly indicates that we are dealing with a system that does not match all competent graduates of Canadian medical schools. The numbers speak for themselves. In 2018, 46 students withdrew after the first iteration of the match. After the second iteration, there were 69 unmatched current year medical graduates, and 54 unmatched prior-year Canadian medical graduates. This total of 169 unmatched graduates is unacceptable.

AFMC published a report on Reducing the Number of Unmatched Canadian Medical Graduates, which includes ten recommendations to significantly resolve the problem. Every stakeholder, from medical schools to government, from program directors to student affairs offices, has a role to play in addressing this situation.

Our undergraduate medical education and student affairs offices have been asked to enhance the support to their students in career counseling, and support for their unmatched graduates. However, significant barriers exist. For example, in some provinces, the medical regulatory authority does not allow unmatched graduates to have access to patients, even under supervision. This hinders the unmatched student’s capacity to maintain their clinical skills and obtain reference letters. Our residency programs and postgraduate deans have been asked to implement a set of recommendations for Best Practice in Admissions and Selection (BPAS) in residency.

However, these are limited measures. The most effective and efficient way to reduce the number of unmatched is to increase the access of our graduates to residency positions. As documented in the AFMC report, the ratio of post grad residency positions to graduates of Canadian medical schools has decreased steadily in the last 10 years from 1:1.1 in 2009 (when we had 11 unmatched) to 1:1.01. Another important factor is that in the second iteration of the match, in many provinces, positions that were originally funded to be available to CMGs are pooled with positions funded for international medical graduates (IMGs).

AFMC is asking Provincial Ministries of Health to consider increasing the number of government funded residency positions and to keep the CMG and IMG positions separate in the second iteration. The AFMC has received endorsement from the medical education community including learner organizations regarding the recommendations of its report.

Faculties of Medicine are committed to continuing all their efforts to increase support to Canadian medical students and to reduce the number of unmatched CMGs. Importantly, we must have reliable data regarding the factors that influence CMG career decision making, the impact of electives, and residency program ranking processes. To address this gap, AFMC is undertaking a comprehensive data strategy, which will identify data on electives and resident program rank order lists that will help us understand the role of electives in residency program decision making.

The AFMC commends the Canadian Federation of Medical Students for their efforts in supporting uCMGs. The support network they have established for the unmatched is a very important resource, and they are uniquely positioned to provide this type of assistance.

As an unmatched CMG, Dr. Persad was very fortunate to find a position in his specialty of choice the year he was unmatched. This is very uncommon. For our current unmatched who are desperately trying to find their way, please know that AFMC will do everything in its realm of influence to improve the system.

